# Membrane protein insertion and assembly by the bacterial holo-translocon SecYEG–SecDF–YajC–YidC

**DOI:** 10.1042/BCJ20160545

**Published:** 2016-09-27

**Authors:** Joanna Komar, Sara Alvira, Ryan J. Schulze, Remy Martin, Jelger A. Lycklama a Nijeholt, Sarah C. Lee, Tim R. Dafforn, Gabriele Deckers-Hebestreit, Imre Berger, Christiane Schaffitzel, Ian Collinson

**Affiliations:** 1 School of Biochemistry, University of Bristol, Bristol BS8 1TD, U.K.; 2 European Molecular Biology Laboratory, Grenoble Outstation, 6 rue Jules Horowitz, Grenoble 38042, France; 3 School of Biosciences, University of Birmingham, Birmingham B15 2TT, U.K.; 4 Arbeitsgruppe Mikrobiologie, Fachbereich Biologie/Chemie, Universität Osnabrück, Osnabrück D-49069, Germany

**Keywords:** holo-translocon, insertion, membrane protein, SecY, YidC

## Abstract

Protein secretion and membrane insertion occur through the ubiquitous Sec machinery. In this system, insertion involves the targeting of translating ribosomes *via* the signal recognition particle and its cognate receptor to the SecY (bacteria and archaea)/Sec61 (eukaryotes) translocon. A common mechanism then guides nascent transmembrane helices (TMHs) through the Sec complex, mediated by associated membrane insertion factors. In bacteria, the membrane protein ‘insertase’ YidC ushers TMHs through a lateral gate of SecY to the bilayer. YidC is also thought to incorporate proteins into the membrane independently of SecYEG. Here, we show the bacterial holo-translocon (HTL) — a supercomplex of SecYEG–SecDF–YajC–YidC — is a *bona fide* resident of the *Escherichia coli* inner membrane. Moreover, when compared with SecYEG and YidC alone, the HTL is more effective at the insertion and assembly of a wide range of membrane protein substrates, including those hitherto thought to require only YidC.

## Introduction

The structure of the conserved heterotrimeric Sec complex lends itself to both protein secretion and membrane protein insertion [1]. Minor rearrangements of the complex, such as the signal sequence-induced displacement of TMH (transmembrane helix)-2b, TMH-7 and a central plug (helix 2a) of SecY/Sec61α, initiate the transport of secretory proteins across the membrane [2,3]. The separation of pseudo-symmetric halves of this subunit is a prerequisite for secretion through the centre of the complex and for the opening of a lateral gate (LG) for membrane protein insertion [1,4,5].

In bacteria, the SecY complex (SecYEG) associates with the motor ATPase SecA, which drives post-translational translocation of pre-proteins across the membrane [6–8]. In contrast, membrane proteins are targeted to the membrane co-translationally by the signal recognition particle (SRP) associating with its receptor at the cytosolic surface [9–12]. The ribosome nascent chain complex is then passed onto the SecYEG complex whereupon protein translocation proceeds; the nascent membrane protein is threaded through the protein channel and onwards into the bilayer *via* the LG. The recently characterised holo-translocon (HTL) super-complex comprises the SecYEG core complex, the accessory sub-complex SecDF–YajC and the so-called membrane protein insertase YidC [9]; in principle, all the components necessary for protein secretion and the insertion of TMHs of translocating membrane proteins.

The analysis of membrane protein insertion is very challenging due to their inherent propensity to associate with membranes. The known tendency of small hydrophobic proteins to spontaneously insert into bilayers may easily be conflated with the controlled and efficient incorporation into membranes by the translocation machinery, required in the cell to avoid aggregation. Until relatively recently, it has long been assumed that the M13 and Pf3 phage coat proteins entered the membrane spontaneously [13,14], a process now known to be dependent on YidC [15]. Based on classical reports that the M13 procoat inserts into the membrane without the assistance of other membrane proteins (including SecY and SecA) [13,16] and on the established sensitivity of Pf3 phage coat protein to the depletion of YidC, it was proposed that this new membrane insertase could act independently of SecYEG [15]. Further support came from the demonstration that the insertion of subunit c of the ATP synthase — F_O_c — was sensitive to the *in vivo* depletion of YidC [17] but not of SecDF, the ancillary sub-complex of the HTL. In addition, *in vitro* insertion of F_O_c was demonstrated to be more efficient into liposomes containing YidC compared with those harbouring SecYEG [18]. Another substrate, the mechano-sensitive channel protein (MscL), is also thought to be inserted by YidC alone, based on its insensitivity to the depletion of the Sec machinery [19].

As far as we know, all other membrane proteins require both SecYEG and YidC for their insertion, a concept exemplified by leader peptidase [20], FtsQ [21,22], CyoA [23] and subunit a of the ATP synthase — F_O_a [17,24]. However, the determinants for the insertion of many membrane proteins have not yet been clarified, including some of those analysed in the present study: EmrE, a multidrug transporter, and GlpG, an intramembrane rhomboid protease.

During the insertion process, YidC is thought to facilitate the passage of nascent TMHs emerging through the LG of SecYEG into the lipid bilayer [22,25]. Atomic structures reveal a cytosolic groove within the membrane, proposed to bind TMHs prior to their propulsion into the bilayer, driven by the membrane potential [26,27]. In this mechanism, TMHs could be received directly from the ribosome (SecY-independent insertion) or *via* the LG of the translocon.

The proposed dependence of different membrane proteins on SecYEG or YidC for insertion based on *in vitro* and *in vivo* results needs to be treated with caution. First of all, membrane protein insertion and folding can occur spontaneously *in vitro* [28]. Secondly, overexpression of membrane protein substrates or the depletion of components of the translocon obviously will have profound and unpredictable effects in the cell — potentially including the misappropriation of membrane proteins.

Therefore, we decided to re-investigate the requirement of membrane protein substrates on the components of the translocation apparatus. The availability of the HTL and the development of a stringent *in vitro* membrane protein insertion assay [9] provide a unique opportunity to verify the fundamental characteristics of the insertion machinery, including the specificity of the HTL, SecYEG and YidC for a range of membrane protein substrates.

## Materials and methods

### Strains, plasmids and antibodies

*Escherichia coli* C43 (DE3) strain and the F_O_c overexpression plasmid [29] were gifts from Sir John Walker (MRC Mitochondrial Biology Unit, Cambridge, UK). *E. coli* JS7131 YidC-depletion strain was a gift from Prof. Andreas Kuhn (University of Hohenheim, Stuttgart, Germany). The SecYEG, YidC and xF_O_c expression plasmids were from our laboratory collections [30–32], and the *E. coli* BL21 strain was purchased from Invitrogen. The HTL expression vector was created using the ACEMBL expression system [9,33].

Mouse monoclonal antibodies against SecY, SecE, SecG and YidC were from our laboratory collection [9,34,35]. Rabbit polyclonal antibodies against SecD and SecF were provided by Prof. Hajime Tokuda (University of Tokyo, Japan). The F_O_c monoclonal mouse IgG (GDH 9-2A2) was raised against SDS-denatured subunit c and recognises the epitope 31-LGGKFLE-37 in the hydrophilic loop [36].

### Preparation of HTL SMA lipid particles

A 20 ml overnight culture of *E. coli* C43 (DE3) in Luria broth was used to inoculate 1 l of 2× YT at 37°C. Once reaching *A*_600_ of 0.8, it was incubated for further 3 h at 37°C. Cells were harvested by centrifugation at 4000×***g*** for 20 min and resuspended in 20 ml of 20 mM Tris–HCl, pH 8.0, 130 mM NaCl and 10% glycerol (TSG). Cells were ruptured using a cell disruptor (Constant Systems Ltd) at 25 kPSI, and the membrane fraction was collected by ultracentrifugation at 148 000×***g*** for 30 min. Membranes were resuspended in 10 ml of TSG buffer per 1 l culture. Styrene maleic acid [SMA, 2.5% (w/v)] copolymer with a 2:1 ratio was added to the membranes and incubated for 2 h rotating at 21°C [37]. Excess membrane was removed by ultracentrifugation at 148 000×***g*** for 45 min, and the supernatant containing SMA lipid particles (SMALPs) was retained for co-immunoprecipitation.

### Co-immunoprecipitation of HTL-SMALP using anti-SecG antibody

Co-immunoprecipitation was performed by incubating 5 µl of monoclonal SecG antibody with 1 ml of SMALP supernatant for 3 h at room temperature (21°C) on a rotary platform. A control sample was prepared without the antibody. The solutions were incubated with 1 ml of protein G (Amintra Protein G, Expedeon), equilibrated in TSG buffer, rotating overnight at 4°C. Samples were centrifuged in 1 ml spin columns (Bio-Rad) at 2000×***g*** for 30 s and washed with 10 column volumes of TSG buffer. Elution was performed with 300 µl of LDS buffer (NuPAGE, Thermo Fisher Scientific) by centrifugation at 3000×***g*** for 30 s. A total of 50 µl of samples were collected and analysed by SDS–PAGE and western blotting for each of the HTL components.

### Protein purification and reconstitution

The HTL, SecYEG, YidC and their cysteine-free variants, SecA and precursor of the outer membrane protein A (proOmpA) were purified as previously described [9,31,38]. The HTL and SecYEG were reconstituted into standard proteoliposomes (PLs) as described [38] using *E. coli* total polar lipids (Avanti Polar Lipids, Inc.). Vesicles were formed by overnight dialysis against a solution of Bio-Beads SM-2 adsorbents (Bio-Rad Laboratories Ltd) in TKM buffer (20 mM Tris–HCl, pH 8.0, 50 mM KCl and 2 mM MgCl_2_), resulting in a final concentration of 4.6 μM SecYEG (2.3 μM active sites) or 2.3 μM of the HTL complex. PLs containing bacteriorhodopsin (bR) were reconstituted using the same method as described [9–12]. YidC standard PLs were prepared using the same method (in the absence of bR) in the presence of YidC reconstitution buffer (20 mM ADA, pH 5.7, 130 mM NaCl) instead of the standard TKM buffer. Briefly, bR-containing purple membranes from *Halobacterium halobium* were purified as described [9,39] and solubilised in the presence of 2% Triton X-100 for 72 h at 21°C. Solubilised bR together with SecYEG, YidC or the HTL was mixed with *E. coli* total polar lipids and hen egg l-α-PC (3:1 ratio, Avanti Polar Lipids, Inc.) in the presence of Triton X-100 in TKM buffer. Detergent was slowly removed by the addition of Bio-Beads (10 mg per 1 mg detergent) every 3 h for 12h, rotating in the dark at 21°C. Formed PLs contained a final concentration of 90 μM bR and 4.6 μM SecYEG or YidC (2.3 μM active sites), or 2.3 μM of the HTL. Proteins were reconstituted assuming a homo-dimeric form of SecYEG and a hetero-dimeric complex of the HTL, consisting of one copy of each subcomplex, SecYEG and SecDF–YajC–YidC [9,13,14]. For the purpose of the reconstitution, YidC was also treated as a homo-dimer. This simplification allowed for equal stoichiometry of the complexes in all sets of PLs, and thus for a direct comparison of their activities. Inner membrane vesicles were prepared as described [34].

### *In vitro* transcription/translation/insertion assay

The membrane protein insertion activity of the HTL, SecYEG and YidC, and their cysteine-free derivatives, was tested as described [9,40]. Briefly, genes of interest were amplified by PCR and transcribed *in vitro* using T7 RNA polymerase for 3 h at 37°C. mRNA was purified using the RNeasy Mini Kit (Qiagen Ltd, Manchester, UK). Generated transcripts were translated using an *E. coli* S-30 cell extract (containing endogenous SecA) [40,41] in the presence of 50 mM Tris acetate (pH 7.5), 14 mM Mg(OAc)_2_, 200 mM potassium glutamate, 30 mM acetylphosphate, 30 mM ammonium acetate, 2 mM ATP, 0.5 mM GTP, 1 mM cAMP, 500 μg/ml tRNA (Type XXI, Strain W), 1.5% PEG 8000, 20 μg/ml folinic acid, 0.2 mM NADH, 2 mM PEP, 0.3–0.5 U pyruvate kinase, 0.45–0.7 U lactate dehydrogenase, 72.5 nM SRP–FtsY complex (single-chain SRP, scSRP) [42], 350 μM each amino acid (except methionine), 40 nM ^35^S-labelled methionine and 0.73 μM (0.37 μM active sites) SecYEG or YidC, or 0.37 μM of the HTL reconstituted into PLs. Reactions were incubated at 21°C for 90 min and translated proteins were co-translationally inserted into PLs. PLs containing inserted substrates were purified by sucrose density gradient centrifugation followed by 5 M urea wash to remove any peripherally bound substrate. Samples were analysed by SDS–PAGE or blue native BN-PAGE. For the purpose of BN-PAGE analysis, samples were solubilised in non-denaturing detergent (DDM, *n*-dodecyl β-d-maltoside), resuspended in 4× NativePAGE sample buffer and loaded onto 4–16% Bis–Tris NativePAGE (Invitrogen). Radiolabelled substrates successfully inserted into the lipid bilayer were detected by phosphor imaging and quantified using ImageQuant.

For the purpose of generating a proton motive force (PMF), bR from *H. halobium* purple membranes was co-reconstituted together with translocation complexes as described [9]. Where bR-containing PLs were used, all reactions were prepared in the dark and incubated in front of a 250 W slide projector fitted with a yellow filter, or in the dark for reactions in the absence of the PMF. To collapse the PMF, an uncoupling ionophore CCCP was used at 50 μM.

### *In vitro* translocation assay

The PMF-stimulated protein secretion activity of the HTL, SecYEG and YidC was tested as described [9] in the presence of 0.3 μM SecA and 0.46 μM (0.23 μM active sites) SecYEG or YidC, or 0.23 μM of the HTL incorporated into phospholipid vesicles for 30 min at 21°C. Following the 30-min incubation, proteinase K was added to all reactions to a final concentration of 0.2 mg/ml followed by a 45-min incubation on ice. To precipitate successfully translocated proOmpA, BSA was added to a final concentration of 1 mg/ml, followed by 20% TCA. After a 30-min incubation, reactions were centrifuged at 4°C for 10 min at 14 500×***g***. Pellets were resuspended overnight in 4× LDS sample buffer and analysed by SDS–PAGE. Protease-protected proOmpA successfully translocated inside the lumen of PLs was analysed by western blotting and the results from three independent repeats were quantified using ImageJ.

### Affinity measurements of YidC to 70S by fluorescence analysis

Cy3-labelled cysteine mutants (60 nM) of YidC in 20 mM Hepes/KOH (pH 7.5), 100 mM KCl, 10 mM MgOAc_2_, 5% (vol/vol) glycerol and 0.1% DDM were mixed 1:1 with 0.6 nM to 19.2 µM 70S ribosomes in 20 mM Hepes/KOH (pH 7.5), 20 mM Mg(OAc)_2_, 30 mM NH_4_Cl and 1 mM dithiothreitol (DTT) in a volume of 25 µl. Samples were incubated for 10 min at 20°C before measurements. Fluorescence was measured using a Monolith NT.115 and data were analysed using the supplied software (Nanotemper). Background fluorescence of fluorescently labelled cysteine-less YidC was subtracted. Each experiment was repeated four times and the fluorescence was normalised by division through the average of the first four data points.

## Results

### Integrity of the HTL

Before embarking on a comprehensive analysis of the activity of the HTL, we thought it prudent to unambiguously establish its existence within native *E. coli* membranes. Previously, a complex of SecYEG–SecDF–YajC was identified in the strain BL21; however, YidC had yet to be identified [43]. Our more recent purification of the recombinant HTL, including YidC, relied on the balanced overexpression of all seven components [9,33]. Therefore, immunoprecipitation experiments were conducted in order to confirm the presence of endogenous intact HTL in the inner membranes of the BL21-derived *E. coli* C43 strain [44].

**Figure 1.**
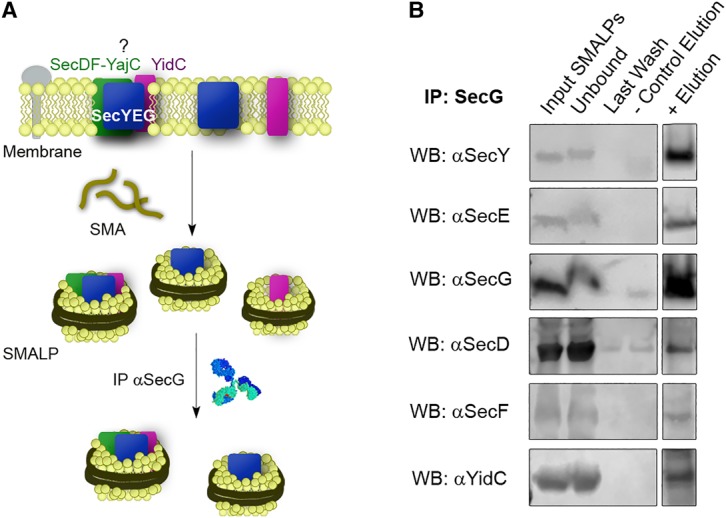
SMA extraction and HTL-SMALP immunoprecipitation. (**A**) SMALP formation. Membrane proteins can be extracted from membranes by the addition of SMA. SMALPs will contain different proteins, for example, the HTL, in their native membrane environment. Immunoprecipitation with a monoclonal antibody α-SecG and Protein G beads will pull only SMALP-HTL and SMALP-SecYEG. (**B**) SMALPs from *E. coli* C43 membranes were immunoprecipitated against SecG antibody and subjected to western blotting against SecY, SecE, SecG, SecD, SecF and YidC. Negative controls in the absence of SecG antibody were also performed (control lanes). Amounts loaded onto the gel: input 10 µl; unbound: 20 µl; last wash: 20 µl; negative control: 50 µl; eluted: 50 µl.

SMALPs provide a novel tool for the extraction of proteins from lipid membranes in the absence of detergents. This method yields disc-shaped particles of ∼10 nm diameter composed of a polymer annulus surrounding an intact core wherein a membrane protein, or complex of them, is surrounded and preserved by native phospholipids (see ref. [37] and references therein). The method eliminates the need to use detergent during any stage of the extraction, allowing for the analysis of protein–protein interactions within membrane protein super-complexes that might otherwise be destabilised by exposure to detergents (Figure 1A) [45–47].

Wild-type *E. coli* (strain C43) total membrane fractions were solubilised using SMA co-polymer and the resulting SMALPs were subjected to co-immunoprecipitation with a monoclonal SecG antibody. Analysis by western blotting against the HTL components (except YajC due to the lack of a specific antibody) revealed the presence of the subunits constituting the HTL (Figure 1B), demonstrating that it is indeed present and intact in native membranes without the overexpression of any component.

### Dependency of membrane protein insertion on the translocon components

Co-translational membrane protein insertion was reconstituted *in vitro* by the addition of mRNA encoding various membrane proteins to an *E. coli* S30 cell extract capable of protein synthesis. The reactions were carried out in the presence of scSRP [42] and PLs containing either HTL, SecYEG or YidC alone. Given that phospholipids are known to stabilise the HTL *in vitro* [9], it is safe to assume the complex is thereby preserved upon membrane reconstitution and retained intact within the resultant PLs.

The HTL is formed by a hetero-dimeric association of the sub-complexes SecYEG and SecDF–YajC–YidC [9], whereas SecYEG alone forms homo-dimers [34,35]. These two different dimers contain a single active translocation site through SecY (the second copy of SecYEG in the homo-dimer is inactive) [2,48]. The oligomeric state and activity of YidC alone is less clear; dimers are also thought to be required for a single active site [31,49]. With this in mind, PLs containing HTL (2.3 µM) were compared with those containing twice the amount of the individual components (4.6 µM; equivalent to 2.3 µM potential active translocation sites).

**Figure 2.**
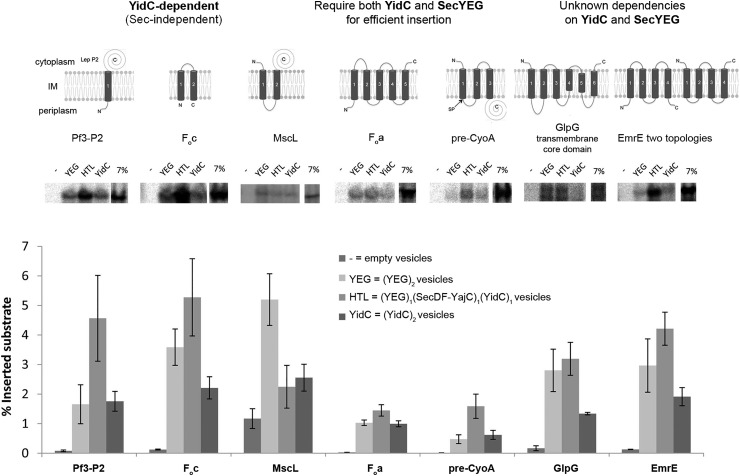
Membrane protein insertion activity of SecYEG, YidC and the HTL. SDS–PAGE analysis of coupled *in vitro* translation/insertion reactions showing membrane incorporation of selected substrates. mRNA was synthesised *in vitro* for selected membrane proteins and subsequently incubated with an *E. coli* S30 cell extract and scSRP [42] in the presence of empty vesicles or PLs containing SecYEG (4.6 μM = 2.3 μM potential active translocation sites), YidC (4.6 μM = 2.3 μM active sites) or the HTL (2.3 μM). Following vesicle floatation and urea treatment, reactions were analysed by SDS–PAGE and successfully inserted radiolabelled protein was detected by phosphor imaging. Quantification was performed using ImageQuant and represents the mean of four independent repeats ± standard error of the mean (SEM). Key explaining PL stoichiometries is presented in the figure.

The reconstitution efficiency of translocation complexes into PLs was assessed by SDS–PAGE, which was consistent between all three samples, SecYEG, YidC and the HTL (Supplementary Figure S1A). Reassuringly, the process of urea extraction, used in the analysis, did not deplete the resident translocon components of the PLs (Supplementary Figure S1B). Therefore, the subsequent results were not unduly affected by variations in the respective concentrations of SecYEG, YidC or the HTL. The yield of *bona fide* membrane protein insertion into the different PLs was then evaluated by vesicle flotation and resistance to urea extraction (Figure 2).

PLs containing the HTL, SecYEG and YidC were challenged with a wide range of nascent membrane proteins including those reputed not to require SecYEG for efficient membrane insertion (Pf3-P2, F_O_c and MscL) and those that do (F_O_a and pre-CyoA), as well as others with unknown translocon dependencies (GlpG and EmrE). With the exception of MscL, the HTL complex proved significantly more effective at membrane insertion of the selected substrates in comparison with SecYEG or YidC individually (Figure 2). Surprisingly, Pf3-P2 and F_O_c were more effectively incorporated into the membrane by the HTL, and MscL by SecYEG, rather than as expected by YidC alone.

The relatively low yield of membrane incorporation (0.5–10%) in these experiments is not a failing of the insertion process *per se*. Rather, it is a consequence of the stringent method employed to distinguish genuine integration into the membrane as opposed to aggregation or non-specific association at the vesicular surface. The main reason for the apparent low insertion efficiency is the uniformly low recovery (∼25%) of the PLs by centrifugation and flotation (Supplementary Figure S1C).

Furthermore, we show that the differences in membrane insertion activity between the HTL, SecYEG and YidC are not a result of variations of the reconstitution efficiency or the orientation within the respective PLs. In each case, the quantities of SecY and YidC and the relative exposure of cytosolic loops to trypsin were shown to be consistent between the HTL and the individual components (Supplementary Figure S2B). The proteolysis was also conducted in the presence of detergent (Triton X-100) in order to dissolve the membrane and expose all the sites to the protease, to give a measure of 100% cleavage for comparison (Supplementary Figure S2B). The analysis shows that the efficiency and orientation of the reconstituted proteins are indeed the same for all three samples: HTL, SecYEG and YidC, and variations in this process did not affect the results.

### The dependence of membrane protein insertion on the PMF

**Figure 3.**
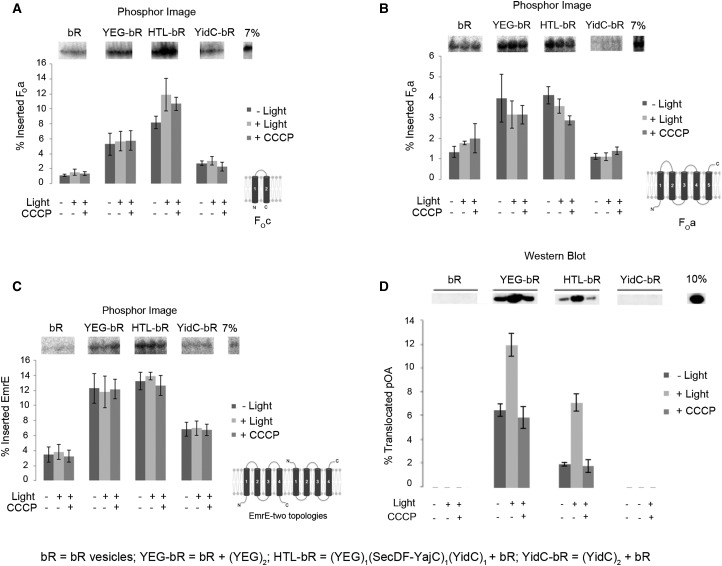
PMF-stimulated activity of the HTL in membrane protein insertion and protein secretion. SDS–PAGE analysis of PMF-stimulated coupled *in vitro* translation/insertion reactions (**A**–**C**) and *in vitro* translocation reactions (**D**), incubated in the presence (+light) or absence (−light) of light, or in the presence of an uncoupling ionophore and light (+CCCP). Successfully inserted radiolabelled F_O_c (**A**), F_O_a (**B**) and EmrE (**C**) or translocated proOmpA (**D**) were detected by phosphor imaging or western blotting, respectively. Quantification was performed using ImageQuant (**A** and **B**) or ImageJ (**C** and **D**), and represents the mean of three independent repeats ± SEM.

Next, we investigated the dependence of substrate insertion on the transmembrane PMF by co-reconstituting the HTL and the individual components SecYEG and YidC into liposomes together with the light-driven proton pump bR. Due to the different lipid composition, the insertion efficiency into bR-containing PLs was uniformly better compared with when standard PLs were used (Figure 2). In agreement with the above results, all substrates tested (F_O_c, F_O_a and EmrE) required SecYEG (alone or within the HTL) for high levels of insertion (Figure 3A–C). YidC alone brought about only background levels of incorporation. Once again, the HTL was most effective for the insertion of F_O_c (Figure 3A and Supplementary Figure S3A). In all cases, there was either only a very minor or no stimulation of the insertion process by the PMF (Figure 3A–C). This is in distinct contrast with the secretion process, monitored by the transport of the proOmpA through SecYEG (in the presence of SecA), which is strongly promoted by the PMF, particularly in the case of the HTL [6,9] (Figure 3D).

**Figure 4.**
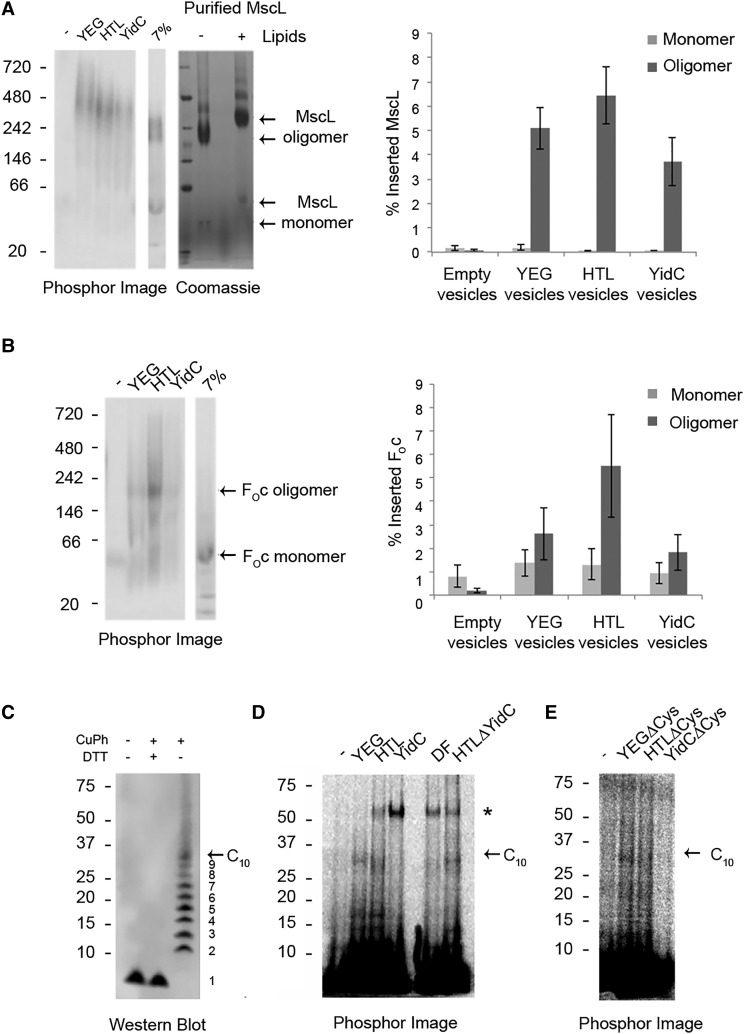
Role of the HTL in membrane protein assembly. BN-PAGE analysis of coupled *in vitro* translation/insertion reactions showing assembly of MscL (**A**) and F_O_c (**B**). Purified MscL was analysed alongside inserted protein in the presence or absence of *E. coli* polar lipids (**A**). Quantification of monomeric and oligomeric species was performed using ImageQuant and represents the mean of three independent repeats ± SEM. (**C**) *E. coli* C43 membranes expressing xF_O_c were analysed by western blotting against F_O_c antibody, in the presence or absence of oxidising (CuPh) and/or reducing (DTT) conditions. (**D**) xF_O_c insertion activity of SecYEG, HTL, YidC, SecDF, HTLΔYidC PLs and (**E**) SecYEG, YidC and HTL Cys-free variants was analysed by SDS–PAGE in non-reducing conditions and detected by phosphor imaging. Arrows indicate oligomeric xF_O_c species and asterisks correspond to unspecific Cys-Cys interactions.

### Membrane protein assembly

Further experiments were conducted to assess the role of the HTL (and resident SecYEG and YidC) in membrane protein assembly. F_O_c and MscL are both naturally occurring homo-oligomers, containing, respectively, 10 [50,51] and 5 [52] subunits. Their assembled state was analysed by BN-PAGE following *in vitro* coupled protein synthesis and membrane insertion. Again, the membrane vesicles were isolated by flotation and subjected to urea extraction, and the inserted membrane proteins were solubilised with a non-denaturing detergent (DDM) and analysed by BN-PAGE. The radiolabelled *in vitro* synthesised and inserted membrane proteins were then visualised by phosphor imaging and compared with those made *in vivo*.

Even though MscL is more efficiently incorporated into the membrane by SecYEG (Figure 2), its assembly is more accurately handled by the HTL, based on the appearance of a more pronounced band corresponding to an oligomeric assembly (Figure 4A, left-hand panel). When YidC or SecYEG was used, the assembled product was more diffuse, possibly due to spurious insertion events leading to increased heterogeneity of the oligomeric states. The higher apparent molecular weight (MW) of the inserted and assembled complex compared with purified MscL is due to its association with lipids, which can be partially replicated by the addition of lipids to the purified material (Figure 4A, right-hand panel).

The assembly of the F_O_c subunit of the ATP synthase could also be monitored by the appearance of a higher MW oligomeric species by BN-PAGE, which again was most effective following insertion by the HTL (Figure 4B).

Additional experiments were carried out to explore the nature of the *in vitro* assembled state of F_O_c. Native *E. coli* ATP synthase forms a decameric ring of subunits (the c_10_-ring), which constitutes the rotary component of the F_O_ membrane domain [32]. The assembled state of the c-ring can be recognised due to its partial resistance to dissociation in SDS–PAGE [29]. The scale of the insertion experiments developed here was increased in order to visualise and characterise the oligomeric state of F_O_c by non-native SDS–PAGE. Faint higher MW bands could indeed be observed following insertion in the presence of SecYEG and the HTL, but not of YidC alone (Supplementary Figure S4A).

Two cysteines (C21 and C65) were introduced into the subunit (referred to hereafter as xF_O_c), which when oxidised form intermolecular cross-links to the adjacent subunit in the c-ring. This strategy was then exploited to determine the stoichiometry of F_O_c in the native complex and the *in vitro* assembled products formed within membranes containing the HTL, SecYEG or YidC.

Exposure of membranes containing *in vivo* incorporated xF_O_c to the powerful oxidant copper-1,10-phenanthroline (CuPh) causes the formation of intermolecular disulphide bonds, which when analysed by SDS–PAGE produce a ladder characteristic of a c_10_-ring with the fully cross-linked form at the top at ∼30 kDa (Figure 4C). The *in vitro* incorporated products were not subjected to extreme oxidation by CuPh in order to minimise any non-specific cross-linking (see below). Nevertheless, the mild oxidising conditions due to the presence of lipids and dissolved atmospheric oxygen were sufficient to generate higher MW cross-links (Figure 4D). In large-scale insertion experiments, PLs containing either the HTL or SecYEG, but not YidC alone, the 30 kDa band corresponding to the fully cross-linked c_10_-ring could be detected along with a few partially cross-linked forms (Figure 4D).

The higher MW band (∼65 kDa) formed in the presence of the HTL and YidC alone, much larger than the cross-linked c_10_-ring, was lost when the cysteines were removed from either the substrate (F_O_c; Supplementary Figure S4B) or the translocon components (Figure 4E). Presumably, this band corresponds to a cross-link between one of the cysteines of the xF_O_c substrate variant and the single native cysteine of YidC or SecD, occurring during the insertion process. This interpretation is consistent with an apparent MW of 65 kDa. This higher MW band formed by only a single disulphide bond between the substrate and one of the translocon subunits was, as expected, more prone to reduction by DTT, compared with the 30-kDa band corresponding to the c_10_-ring linked together by 10 disulphides (Supplementary Figure S4B).

Interestingly, significant amounts of the cross-linked c_10_-ring can also be detected following insertion conducted even in the absence of YidC (Figure 4D). In these experiments, a 65-kDa band was again observed, confirming that the substrate variant xF_O_c can be also cross-linked to either YidC or SecD.

The significance of the higher MW cross-link at this stage cannot be ascertained. However, the overriding findings from the analyses on the assembly of MscL and the c_10_-ring of F_O_ show an absolute requirement for SecYEG for complex assembly in a process further facilitated by the SecDF–YajC–YidC sub-complex.

### Interaction of the translocon with the ribosome

**Figure 5.**
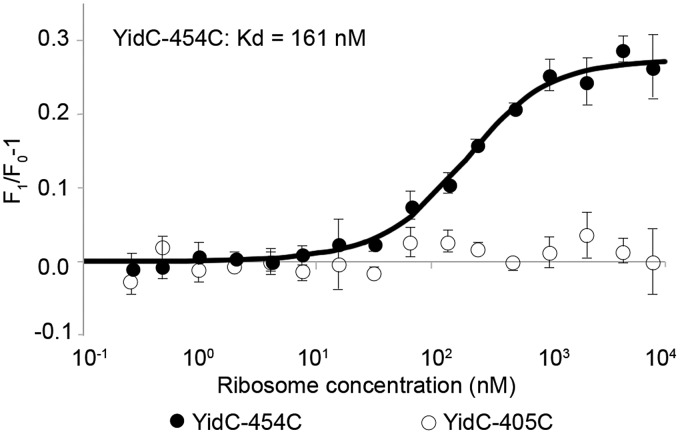
Interaction of YidC with the ribosome. Binding of detergent-solubilised YidC to 70S ribosomes followed by fluorescence intensity. Constant amounts of YidC labelled with Cy3 at positions 405 or 454 were exposed to increasing concentrations of 70S ribosomes, leading to an increased fluorescence in the case of YidC labelled at position 454, but not at 405. The results represent the mean of four independent experiments. The standard deviation of the average *K*_d_ value for YidC (161 nM) is ±36 nM.

Membrane protein insertion is thought generally to occur during translation. Given that the HTL is most effective at the insertion and assembly process, we expected that its interaction with the ribosome would be more favourable than with individual subcomplexes, which indeed proved to be the case. The HTL displays a higher ribosome-binding affinity compared with SecYEG [9]. Using the method we described recently [9], the affinity of detergent-solubilised YidC for 70S ribosomes was determined by fluorescence analysis of YidC labelled with Cy3 at positions 454 (periplasmic side) and 405 (cytoplasmic side). The affinity of YidC for ribosomes was determined by the increase in Cy3 fluorescence at position 454 upon binding (Figure 5). No increase in fluorescence was observed in the case of YidC labelled at position 405, used as a control. Interestingly, the affinity of YidC for 70S ribosomes (161 nM) is weaker than that of the HTL (35 nM), and similar to SecYEG alone (200 nM) [9]. This higher affinity correlates with the improvement in insertion activity and suggests that SecYEG and YidC both contribute to the functional interface with the ribosome.

## Discussion

The molecular mechanism underlying membrane protein insertion, folding and assembly is poorly understood compared with what we know about the transport of proteins across the membrane during secretion [8,53]. The availability of the bacterial machinery — the HTL [9,33] — capable of both secretion and membrane insertion has provided a unique opportunity to address this problem. Crucially, we show here that the HTL complex can be extracted intact from the plasma membrane of wild-type *E. coli* as SMALPs, suggestive of its existence as a stable entity within native membranes. This important verification substantiates our previous [9] and present analysis of the HTL produced by recombinant overexpression.

The classification of F_O_c, Pf3-P2 and MscL as substrates that require only YidC for membrane insertion, independent of SecYEG, is based largely on *in vivo* depletion studies [17,19,54,55]. However, this approach is known to trigger secondary effects in the membrane composition of depleted cells [54,56,57]. One way to overcome this problem is to monitor membrane protein insertion reconstituted from purified components, which we achieved using two completely independent reconstitution methods (standard PLs and bR-PLs) employing different detergents and lipids. Our *in vitro* analysis of membrane proteins thought to require both SecY and YidC, as expected, has shown that all substrates are inserted and assembled most efficiently by the HTL. However, the results also show that those membrane proteins previously designated to be solely dependent on YidC for their insertion (see Introduction) were, in fact, more efficiently incorporated and assembled by the HTL, compared with YidC or SecYEG individually.

Small hydrophobic membrane proteins, such as F_O_c used in the present study, spontaneously insert into lipid bilayers [58]. We also observe this non-specific process in the absence of any translocon component (lipids alone or PLs containing only bR; Figures 3A and 4D,E). However, insertion activity of the HTL is considerably higher compared with this background. Moreover, the HTL-inserted F_O_c and MscL result in the assembly of the native oligomeric states, matching those formed *in vivo.* Therefore, these proteins are more faithfully incorporated and folded by the combined actions of SecYEG and YidC present in one super-complex — such that they enter the membrane in a non-aggregated assembly competent conformation.

The data indicate that SecYEG, but not YidC, is an essential factor in the process of membrane protein insertion and assembly. Nevertheless, the SecDF–YajC–YidC sub-complex seems to facilitate this process within the confines of the HTL; presumably, this is attributable to the activity of YidC [22,59]. Unlike SecA-driven translocation through the membrane [9], the PMF does not appear to have a significant stimulatory effect on the insertion of membrane proteins. However, we do not exclude the possibility that the insertion of other membrane proteins by the HTL may be more substantially promoted by the action of the PMF. For instance, substrates requiring the secretion of large periplasmic loops across the membrane may require the action of SecA, which is subjected to PMF stimulation (Figure 3D) [6,9].

Evidence supporting the existence of an insertion pathway utilising only YidC for selected membrane proteins arises from studies of strains depleted in the YidC protein [15,17,19,54,55]. However, this depletion process obviously triggers pleiotropic effects, in particular in respect of membrane composition. The depletion of YidC has severe consequences on the well-being of the cell due to the reduced efficiency in the insertion of essential membrane proteins, including F_O_c [17,54], and presumably also SecY, SecE and SecG. As a consequence, following the depletion of YidC, we could hardly detect any SecY at all (Supplementary Figure S5). Therefore, the insertion and assembly of F_O_c cannot be safely attributed to the activity of YidC alone.

The *in vitro* experiments presented here suggest that SecYEG is indeed required for effective insertion of the membrane protein substrate F_O_c, amongst others previously thought to do so in its absence. This process presumably occurs at the interface between the LG of SecY and YidC [25]. In this scenario, the *in vivo* depletion of YidC, and consequently SecY, would be catastrophic. This could therefore explain the different conclusions drawn from *in vitro* experiments using a pure reconstituted system (present study) compared to the traditional view of YidC functioning as an independent insertase, drawn from *in vivo* analyses of membrane protein insertion [15,17,19,54,55].

Previous estimations of YidC abundance based on ‘semi-quantitative’ western blotting were ∼10 times higher than SecY and SecE [60,61], implying a role independent of the SecY complex. However, more reliable values for the relative amounts of proteins in the *E. coli* proteome are now available [62] that accurately reflect the stoichiometry of membrane protein complexes of known structure; for instance, SecYEG and the F_O_ domain of the ATP synthase (Supplementary Table S1). In the present study, the relative abundance of the components of the HTL — SecYEG:YidC:SecDF — turns out to be ∼4:3:1 (Supplementary Table S1). Therefore, there may not be any excess YidC at all. Low proportions of SecDF suggest the HTL might not be the only version of the translocon. As far as we know, complexes of SecYEG–YidC are not stable in detergent solution, but may be formed in the native membrane environment.

**Figure 6.**
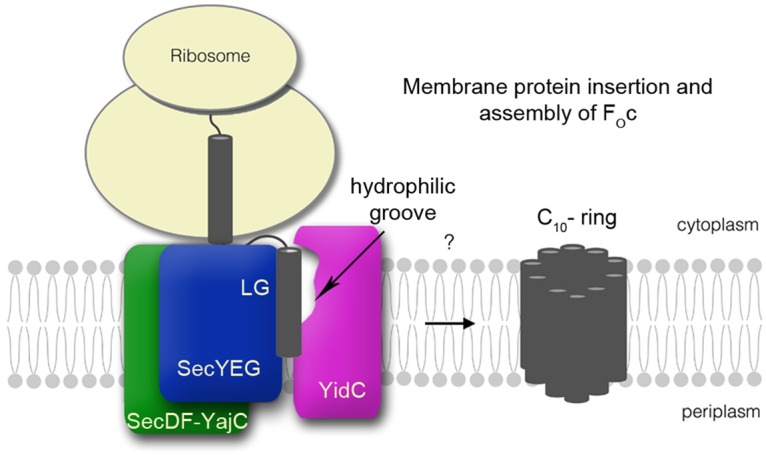
Proposed role of YidC in membrane protein insertion and assembly. The hydrophilic groove of YidC positioned in close proximity to the LG of SecY helps escort TMHs to the bilayer. The resulting shuttle from the protein channel in SecYEG to YidC and the membrane might facilitate efficient folding, insertion and assembly of membrane proteins, for example F_O_c.

In light of the results shown here, the classical view of YidC acting as an independent translocase may need to be reconsidered. A cytosolic cavity that has been identified in the structure of YidC, thought to channel TMHs into the bilayer, has been shown to play a role in membrane insertion of MifM [27]. This resulted in a proposed model for insertion of single spanning membrane proteins displaying a negatively charged periplasmic domain, limited however to only one type of substrate [27]. Perhaps, this cytosolic channel in YidC receives TMHs emerging from the LG of SecY within the HTL. The juxtaposition of the LG and cytosolic cavity of YidC could conceivably give rise to a consolidated TMH shuttle from the protein channel of SecYEG *via* the LG to YidC (and possibly SecDF), and then to the membrane, thereby facilitating efficient folding, insertion and assembly (Figure 6). Similar mechanisms may exist in eukaryotes to promote efficient membrane protein folding and assembly, for example, at the interface between Sec61 and ancillary factors, such as TRAP [63].

Structures of SecYEG [1], SecDF [64] and recently of YidC [26] provide important clues about protein secretion and membrane protein insertion. Our results point to architectural principles, which cannot be explained by the isolated structures. The mechanism of membrane protein insertion has been addressed within the context of the super-molecular assembly of these constituents within the HTL, in particular at the co-operative interface between SecY and YidC.

The availability of an intact HTL complex was made possible by the recent development of the ACEMBL technology, which provides means of flexible and rapid production of multi-gene expression constructs [33,65]. Thus, it is now possible to further explore the structure, dynamics and activity of the HTL and thereby decipher the molecular mechanism of membrane protein insertion through the Sec machinery, and by analogy through other translocons, such as those found in mitochondria and chloroplasts.

## Abbreviations

ADA, *N*-(2-Acetamido)iminodiacetic acid; BN, blue native; bR, bacteriorhodopsin; CCCP, carbonyl cyanide 3-chlorophenylhydrazone; CuPh, copper-1,10-phenanthroline; DDM, *n*-dodecyl β-d-maltoside; DTT, dithiothreitol; HTL, holo-translocon; LDS, lithium dodecyl sulfate; LG, lateral gate; MW, molecular weight; PEP,phosphoenolpyruvate; PL, proteoliposome; PMF, proton motive force; proOmpA, precursor of the outer membrane protein A; SRP, signal recognition particle; scSRP, single-chain SRP; SEM, standard error of the mean; SMA, styrene maleic acid; SMALP, SMA lipid particle; TCA, trichloroacetic acid; TMH, transmembrane helix; TRAP, translocon-associated protein.

## Author Contribution

J.K., S.A., R.J.S., R.M., J.A.L.a.N., I.B., C.S. and I.C. designed research. J.K., S.A., R.M., J.A.L.a.N., C.S. and I.C. performed experiments and analysed data. J.K., S.A., R.M. and J.A.L.a.N. prepared figures. J.K., S.A., R.J.S, I.B., C.S. and I.C. wrote the manuscript.

## Funding

This work was supported by a doctoral training grant from the Biotechnology and Biological Sciences Research Council (BBSRC) (J.K.), a University of Bristol Postgraduate Scholarship (R.M.), BBSRC Project Grants [BB/M003604/1 (S.A. and I.C.) and BB/F007248/1 (R.J.S. and I.C.)], a BBSRC/EPSRC Synthetic Biology Research Centre grant (BrisSynBio) [BB/L01386X/1 (J.K. and I.C.)], an European Research Council (ERC) Starting Grant Award [281331 (C.S.)], a European Commission Framework Programme 7 ComplexINC project [279039 (I.B.)], a European Molecular Biology Organisation Long-Term Fellowship (EMBO LTF) [ALTF 710-2015; (S.A.)] and a European Commission Marie Curie Actions [LTFCOFUND2013, GA-2013-609409; (S.A.)].
